# Temporal Dynamics and Disturbance Responses in Coral‐Dwelling Decapods Provide a Novel Perspective on Their Ecological Role in Coral Reef Systems

**DOI:** 10.1002/ece3.71474

**Published:** 2025-06-03

**Authors:** Susanne Bähr, Natalie Dunn, Sancia E. T. van der Meij, Joydeep Chowdhury, Francesca Benzoni

**Affiliations:** ^1^ Marine Science Program, Biological and Environmental Science and Engineering Division (BESE) King Abdullah University of Science and Technology (KAUST) Thuwal Saudi Arabia; ^2^ Groningen Institute for Evolutionary Life Sciences (GELIFES) University of Groningen Groningen the Netherlands; ^3^ Naturalis Biodiversity Center Leiden the Netherlands; ^4^ Statistics Program King Abdullah University of Science and Technology (KAUST) Thuwal Saudi Arabia

**Keywords:** coral bleaching, Cryptochiridae, fate‐tracking, functional diversity, population dynamics, symbiosis

## Abstract

Symbiotic relationships between corals and invertebrates contribute significantly to coral reef biodiversity. However, their ecological functions within this ecosystem remain understudied due to limited knowledge of the interplay among lifehistory strategies, host density and condition, population variations, and mortality rates. To address this, we investigated the population dynamics of coral‐dwelling gall crabs (Cryptochiridae), obligate symbionts of scleractinian corals, across four central Red Sea reefs. Combining transect surveys with a novel fate‐tracking approach, we monitored 799 crabs on 517 host colonies from September 2022 to 2024. Our data revealed significant variation in host community composition, with reef‐specific conditions shaping crab abundance and diversity more than cross‐shelf gradients. Fate‐tracking uncovered unexpectedly frequent crab colonization and extinction events and a strong preference for settling on already inhabited hosts. In 2023, a mass reef bleaching event provided a unique opportunity to assess disturbance impacts one year into our study, resulting in greater population declines on inshore reefs. Interestingly, fate‐tracking showed that most sites maintained reproductively active crab populations despite bleaching, while compounded stressors at one site caused a local population collapse. Our findings underscore the complex dynamics of the relationship between cryptochirids and their coral hosts, where high reproductive output may offset the costs of host specificity and settlement requirements, thus enabling resilience to moderate disturbances. This study provides novel insights into cryptochirid ecology, revealing unexpectedly high temporal variability in their populations. The observed dynamics suggest gall crabs may occupy a functional role akin to cryptobenthic reef fish by contributing to reef energy transfer, converting host‐derived resources like coral mucus into forms accessible to higher trophic levels and supplementing zooplankton communities with larvae. In light of increasing disturbances, this study highlights the need to integrate reef invertebrates into coral reef conservation strategies for preserving biodiversity and sustaining ecosystem functionality in a rapidly changing world.

## Graphical abstract

Our study investigates the temporal dynamics and disturbance responses of coral‐dwelling gall crabs (Cryptochiridae), an overlooked yet ecologically significant group of obligate coral symbionts. By integrating transect surveys with a novel fate‐tracking approach, we reveal unexpectedly high turnover rates in gall crab populations, allowing us to hypothesize about their ecological significance. A coinciding mass reef bleaching event enabled us to assess their vulnerability to environmental disturbances, highlighting both their resilience through high reproductive output and their dependence on host coral survival, with broader implications for reef trophic dynamics and conservation.

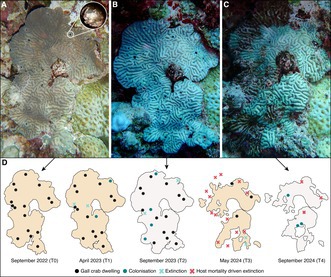

## 1 Introduction

Symbiotic relationships are a cornerstone of the coral reef ecosystem (Apprill 2020). Scleractinian corals rely on symbioses with Symbiodiniaceae and diverse microbial communities, which together constitute the coral holobiont, enabling corals to act as primary reef builders (Muller‐Parker et al. 2015; Pogoreutz et al. 2020; Robinson et al. 2024). Beyond this interaction, corals provide habitat for numerous cryptic invertebrates, many of which are obligately dependent on their host for food and shelter (Stella, Pratchett et al. 2011; Castro 2015). While some of these symbionts, such as *Trapezia* Latreille, 1828 crabs, offer ecological services that enhance coral resilience (Rouzé et al. 2014 and references therein), the roles of many coral‐associated invertebrates remain understudied (Montano 2022). A lack of data on fundamental biological parameters—such as settlement behavior, lifespans, mortality rates, and how host dependency shapes symbiont life history strategies—hinders our understanding of how these symbionts contribute to the movement and storage of energy on the reef, ultimately defining their ecological function (Bellwood et al. 2019). Given their cryptic nature, identifying measurable traits to address these gaps is challenging.

**FIGURE 1:**
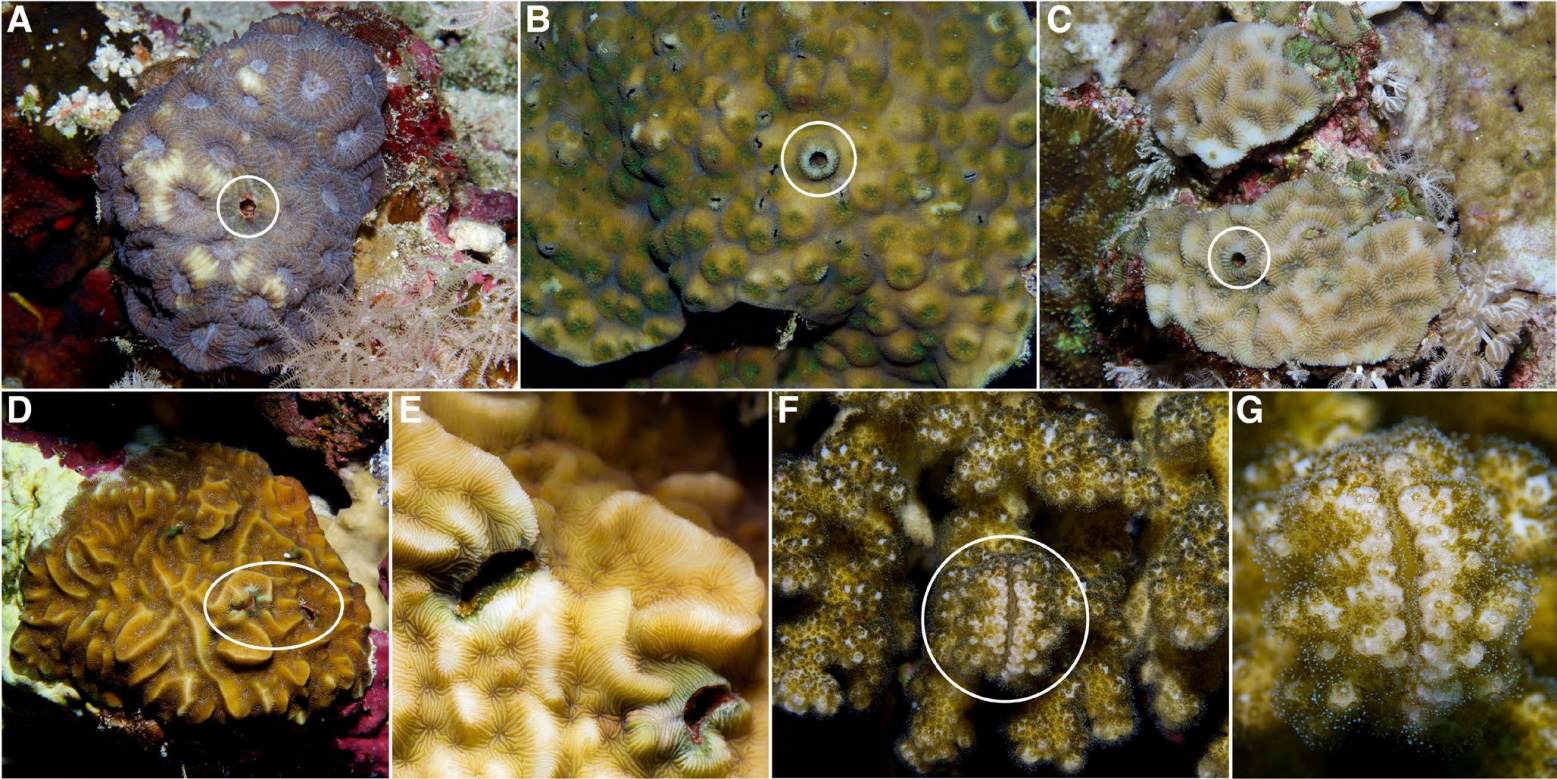
In situ photographs of the most frequently encountered host coral genera and their associated gall crabs. (A) *Dipsastraea* sp. with a cylindrical pit inhabited by *Lithoscaptus* sp. (B) *Lithoscaptus* sp. in its pit within *Echinopora gemmacea* . (C) *Goniastrea pectinata* with a pit of *Lithoscaptus* sp. (D) *Pavona* cf. *varians* with two canopy‐shaped tunnels inhabited by *Opecarcinus* sp. (E) Enlarged view of *Pavona* cf. *varians* with two *Opecarcinus* dwellings. (F) *Pocillopora* favosa with *Hapalocarcinus marsupialis* s.l. dwelling in an enclosed gall formed by host colony branches. (G) Enlarged view of the same *Hapalocarcinus* gall (F). White ellipses mark the dwellings in the zoomed‐out colony photographs.

Broad ecological concepts, like the r/K‐selection framework, provide a valuable lens for understanding lifehistory strategies across ecological gradients, with r‐strategists thriving in unstable environments through high reproduction, while K‐strategists optimize survival and resource use in stable ecosystems (Pianka 1970). However, symbiotic relationships complicate this dichotomy as symbiont life histories depend on host availability and condition, while hosts are influenced by their symbionts' roles (Denison and Kiers 2011). For example, gall crabs (Cryptochiridae), obligate coral‐dwelling decapods, combine r‐strategist traits like high fecundity with K‐strategist traits like strong host specificity, residing in highly specialized skeletal modifications of their hosts (Figure 1) (Wei et al. 2013; van der Meij and Schubart 2014; Bähr et al. 2021). Indeed, the r/K‐selection framework can oversimplify systems where density‐dependent factors play a role (Reznick et al. 2002). This may be especially relevant for obligate coral‐dwellers, where host availability likely influences lifehistory strategies and population dynamics.

At the reef scale, gall crabs typically inhabit around 20% of available coral hosts (van Tienderen and van der Meij 2016 and references therein), even though they can reach high local densities of up to 200 individuals per m^2^ (Hoeksema and van der Meij 2013). This discrepancy suggests a complex interplay among host specificity and availability, life history strategies, and other ecological factors, such as settlement success and environmental conditions, in shaping population dynamics. Given the limited knowledge of these processes, studying temporal variations in gall crab populations, such as individual turnover on host colonies, may offer a chance to uncover these relationships and their broader ecological importance.

Since the 1950s, global reef cover has halved due to rising sea surface temperatures, ocean acidification, pollution, and overfishing (Knowlton 2001; Sully et al. 2022). Intensifying coral bleaching events are driving mass mortality, with nearly all reefs projected to face severe threats by 2050 (Burke et al. 2011). These pressures not only jeopardize coral biodiversity but may also trigger cascading co‐extinctions for symbionts reliant on (specific) coral hosts (Stella, Pratchett et al. 2011). To date, most research on coral‐associated fauna has emphasized community‐level dynamics (e.g., Salas‐Moya et al. 2021; Rhoades et al. 2023), with limited attention given to the population variability of individual obligate coral‐dwelling taxa. This oversight is concerning, given the global decline of coral reefs (Souter et al. 2021).

This study aims to fill critical gaps in our understanding of coral‐dwelling invertebrate ecology by combining baseline transect surveys with a novel fate‐tracking approach to investigate population dynamics of gall crabs across four coral reefs in the central Red Sea (RS). Adapted from coral demography studies, where fate‐tracking is widely used to assess survival after settlement or colony‐level responses to stressors such as bleaching and disease (e.g., Sarribouette et al. 2022; Neely et al. 2022), this method is applied here for the first time to an obligate coral‐dwelling symbiont. It allows detection of settlement, extinction, and mortality events at the individual level, providing fine‐scale temporal resolution on symbiont population dynamics. From September 2022 to September 2024, transect surveys (static snapshots of host and crab occurrence) were conducted to characterize host community composition, gall crab abundance, and prevalence. In addition, fate‐tracking quantified temporal variation, revealing turnover rates through time. One year into the study (September 2023), the occurrence of a mass bleaching event provided an unprecedented opportunity to assess the effects of environmental disturbances and host mortality on both host communities and their symbionts, offering broader insights into the interplay between coral health and gall crab population dynamics.

Specifically, this study seeks to answer the following questions: (1) What factors drive gall crab population dynamics across different host communities? (2) How do baseline surveys and fate‐tracking compare in capturing turnover rates? While transect surveys provide a broad‐scale assessment of host and crab occurrence, fate‐tracking is expected to reveal greater temporal variability, better reflecting spatial heterogeneity in host composition, density, and cross‐shelf gradients. (3) How does disturbance, such as coral bleaching, influence gall crab populations and their turnover? Given that environmental conditions and host availability are key drivers of symbiont survival, reefs with lower bleaching severity and higher host coral densities are expected to support more stable cryptochirid populations, whereas degraded reefs may experience increased local extinction and reduced larval recruitment. By answering these questions, this study provides novel insights into cryptochirid ecology and examines ecological implications of symbiont‐host dynamics in coral reef ecosystems.

## 2 Methods

### 2.1 Study Design

The study took place in the central RS at four reefs encompassing different environmental conditions and distances from shore ranging from 5 to 20 km (Figure S1), with all surveys conducted using SCUBA diving. Nearshore reefs in the study area are characterized by slower‐growing coral taxa, increased turf algal cover, higher turbidity, and elevated temperature regimes, while offshore reefs support more structurally complex and diverse coral assemblages in clearer conditions (Khalil et al. 2017). Coral host and associated gall crab monitoring spanned 2 years, with semiannual surveys at five time points: September 2022 (T0), April 2023 (T1), September 2023 (T2), May 2024 (T3), and September 2024 (T4). At T0, three replicate permanent belt transects (0.5 × 20 m) were established at each study site between 5 and 10 m depth, as part of a broader ongoing coral monitoring effort led by the second author. This semiannual frequency was chosen as a biologically reasonable interval for detecting short‐term changes in gall crab presence based on the best available knowledge at study onset. The study consisted of two complementary and independently analyzed surveys conducted within the same permanent transects: (1) fate‐tracking of tagged coral colonies and their associated gall crabs, and (2) baseline surveys of the entire host and associated cryptochirid community.

### 2.2 Fate‐Tracking of Tagged Colonies and Associated Cryptochirids

Concurrent with transect setup at T0, we tagged 517 coral colonies (both inhabited and uninhabited) of known RS cryptochirid host genera for fate‐tracking (van der Meij et al. 2015). Tagged colonies were evenly distributed within the belt transect area and selected randomly, with no preference given to colony size, location, or prior occupation by gall crabs. To ensure representative coverage, tagged colonies were chosen to reflect an even distribution among the most abundant host genera: *Pocillopora*, *Pavona*, *Echinopora*, *Goniastrea*, *Dipsastraea*, and *Platygyra*, to allow consistent sampling across different reefs. Each tagged colony was revisited at all five time points (T0–T4). At each time point, the maximum colony diameter was recorded. When partial colony mortality occurred, the size of the distinct surviving portions was categorized into small (1–15 cm), medium (16–30 cm), large (31+ cm), or mixed, if multiple fragment sizes were present on the same colony. Each coral colony's health condition was categorized as paling (uniform loss of pigmentation), partially bleached (distinct white patches alongside pigmented tissue), fully bleached (≥ 95% of the colony surface bone white), fluorescent, or showing partial fresh mortality, following thresholds adapted from Jones (2008). Full mortality (recent or old) was recorded for colonies that died between consecutive time points. The presence and number of inhabited gall crab dwellings were noted for each coral colony. Photographs were taken around the entire colony to record their position and serve as a permanent reference for confirming and counting dwellings for crab fate‐tracking.

### 2.3 Baseline Surveys

Baseline surveys were conducted at T1 and T3 to characterize the full coral host community and associated cryptochirid populations within each belt transect. These surveys included all potential gall crab host colonies within the transect area, which also included the previously tagged colonies. All colonies were examined using the same protocol applied during fate tracking. For each colony, we photographed all sides and recorded maximum diameter or fragmentation class, health condition, visible recent or old mortality, and the number of inhabited gall crab dwellings. Baseline surveys were conducted in spring (T1 and T3) to avoid peak thermal stress and potential coral bleaching events, which typically occur in late summer or autumn in the study area (Monroe et al. 2018). Surveys were time‐based, with 1 h allocated per transect. Transects with high diversity or many fragmented and/or small‐sized colonies would have required additional time to fully survey. Consequently, the surveyed belt transect area varied by site (Table S1). Statistical analysis of community composition was therefore conducted on relative abundances of host colonies. To minimize bias, the same surveyor conducted all surveys throughout the study. For the baseline surveys, prevalence rates of gall crabs were calculated as the proportion of inhabited host colonies per transect. Throughout the manuscript, data from fate‐tracked colonies and baseline surveys were analysed and presented separately.

The corals were visually identified to the lowest taxonomic level in situ (Al Tawaha et al. 2019). No gall crab sampling was performed for species‐level identification to avoid disturbing the monitored associations during the study period. Cryptochirids were therefore identified to the lowest taxonomic level based on prior knowledge of their host specificity and dwelling morphology, as adopted in other transect‐based studies of cryptochirids (e.g., van Tienderen and van der Meij 2016) (Table 1). Genus‐level identification was ambiguous in four cases due to the co‐occurrence of three (also Mykescola, Fungicola now) genera on the same host (denoted in Table 1), but as no genus‐level comparisons were made, this did not affect downstream analyses.

**TABLE 1:** Overview of known coral host genera and their associated gall crab genera (with taxonomic authorities provided) previously recorded in the Red Sea, including whether the host genera were observed during baseline surveys and if they were inhabited by gall crabs.

Host taxon	Observed	Crab genus	Association observed
Agariciidae Lamarck, 1801			
*Gardineroseris* Scheer & Pillai, 1974	y	*Opecarcinus* Kropp & Manning, 1987	y
*Leptoseris* Milne Edwards & Haime, 1849	y	*Opecarcinus*	y
*Pavona* Lamarck, 1801	y	*Opecarcinus*	y
		*Pseudohapalocarcinus* Fize & Serène, 1956 (restricted to *Pavona danai* (Milne Edwards, 1860) in the Red Sea)	y
Dendrophylliidae Gray, 1874			
*Turbinaria* Ehrenberg, 1834	y	*Neotroglocarcinus* Takeda & Tamura, 1980	n
Fungiidae Dana, 1846			
*Ctenactis* Verrill, 1864	y	*Fungicola* Serène, 1968	n
*Cycloseris* Milne Edwards & Haime, 1849	y	*Mykescola* van der Meij, 2025	n
*Fungia* Lamarck, 1801	y	*Fungicola*	y
*Herpolitha* Eschscholtz, 1825	y	*Mykescola Fungicola*	n
*Pleuractis* Verrill, 1864	y	*Mykescola*	n
*Podobacia* Milne Edwards & Haime, 1849	n	*Mykescola Fungicola*	n
Leptastreidae Rowlett, 2020			
*Leptastrea* Milne Edwards & Haime, 1849	n	*Dacryomaia* Kropp, 1990	—
Lobophyllidae de Blainville, 1830			
*Acanthastraea* Milne Edwards & Haime, 1848	y	*Fizesereneia* Takeda & Tamura, 1980	y
*Echinophyllia* Klunzinger, 1879	y	*Xynomaia* Kropp, 1990	y
*Lobophyllia* de Blainville, 1830	y	*Fizesereneia*	y
*Oxypora* Saville Kent, 1871	y	*Xynomaia*	y
Psammocoridae Chevalier and L. Beauvais, 1987			
*Psammocora* Dana, 1846	y	*Dacryomaia*	y
Merulinidae Milne Edwards and Haime, 1857			
*Astrea* Lamarck, 1801	y	*Lithoscaptus* A. Milne‐Edwards, 1862	n
*Dipsastraea* Blainville, 1830	y	*Lithoscaptus* *Sphenomaia* Kropp, 1990	y*
*Echinopora* Lamarck, 1816	y	*Lithoscaptus*	y
*Favites* Link, 1807	y	*Lithoscaptus*	y
*Goniastrea*, Milne Edwards & Haime, 1848	y	*Lithoscaptus*	y
*Hydnophora* Fischer von Waldheim, 1807	y	*Lithoscaptus* *Hiroia* Takeda & Tamura, 1981	y
*Leptoria* Milne Edwards & Haime, 1848	n	*Lithoscaptus*	—
*Merulina Ehrenberg, 1834*	y	*Lithoscaptus*	y
*Mycedium* Milne Edwards & Haime, 1851	y	*Xynomaia*	n
*Oulophyllia* Milne Edwards & Haime, 1848	y	*Lithoscaptus*	y
*Paramontastraea* Huang & Budd, 2014	y	*Sphenomaia*	y
*Platygyra* Ehrenberg, 1834	y	*Lithoscaptus* *Cryptochirus* Heller, 1860	y*
Pocilloporidae Gray, 1840			
*Pocillopora* Lamarck, 1816	y	*Hapalocarcinus* Stimpson, 1859	y
*Seriatopora* Lamarck, 1816	y	*Hapalocarcinus*	n
*Stylophora* Schweigger, 1820	y	*Hapalocarcinus*	n
Plesiastreidae Dai and Horng, 2009			
*Plesiastrea* Milne Edwards & Haime, 1848	y	*Lithoscaptus*	—

*Note:* Asterisks denote instances where multiple gall crab genera co‐occur on a single host taxon and cannot be visually distinguished in situ. Data curated from van der Meij et al. 2015, Bähr et al. 2021, Xu et al. 2022, Claassen et al. 2024 and Bähr et al. 2025. A new gall crab genus, *Mykescola*, was recently described to accommodate two species previously classified in *Fungicola* (van der Meij 2025).

### 2.4 Fate‐Tracking and Turnover Calculations

All turnover calculations were based on the fate‐tracking dataset derived from tagged coral colonies, which were surveyed across all five time points (T0–T4). Since turnover captures change between consecutive time points, we refer to each interval as a transitional period (TR1 = T0–T1, TR2 = T1–T2, etc.). Three key events were identified within the first 6 months and formed the basis of subsequent analysis: (1) colonization (establishment of a new dwelling), (2) extinction (when a dwelling was no longer visible on the host surface), and (3) host mortality‐driven extinction (when partial or full mortality of the host colony caused the death of the associated crab, leaving the dwelling visible in the host skeleton). Extinction events, hereafter referred to only as extinctions describe individual‐level processes rather than local or species‐level extinctions. Gall crab turnover was calculated based on colonization and extinctions for each individual colony based on Buckley et al. (2021):

$$\text{Turnover}=\frac{\left(\text{Extinctions}+\text{Colonizations}\right)}{\left(\text{Dwellings}\;T_{\text{Previous}}+\text{Dwellings}\;T_{\text{Current}}\right)}\times 100$$

Additionally, background mortality (the loss of crabs due to factors unrelated to host mortality) and disturbance‐based mortality (the loss of crabs due to host colony mortality) rates were calculated. Both mortality rates were calculated by dividing the number of crabs lost in each manner by the total number of crabs present in the colony at the previous time point.

### 2.5 Environmental Data

Water temperature loggers (Star‐oddi DST CT, Star‐Oddi Garðabær, Iceland) were deployed at each transect at T0, recording temperatures at 10‐min intervals (30 min intervals from T3 onward). To assess thermal stress, we retrieved daily sea surface temperature (SST) data at 5 km resolution for the closest NOAA station (Makkah/Medinah; NOAA Coral Reef Watch 2023). The Maximum Monthly Mean (MMM) temperature for the region was determined as 30.9°C, with a bleaching threshold of 31.9°C based on NOAA climatology. Using the MMM, Degree Heating Week (DHW) values were calculated from in situ temperature data. DHWs quantify cumulative thermal stress over a 12‐week period, calculated as the sum of positive temperature anomalies ≥ 1 °C above the MMM. Higher DHW values are associated with increased risk of coral bleaching and mortality (Liu et al. 2014).

### 2.6 Statistical Analyses

All statistical analyses were performed in R (v4.4.2; R Development Core Team 2024), using the packages *vegan* (Oksanen et al. 2013), *gamlss* (Rigby and Stasinopoulos 2005), *lubridate* (Grolemund and Wickham 2011), and *xts* (Ryan and Ulrich 2011). A Shapiro–Wilk test was conducted to assess normality for all analyses requiring a normality assumption, and Levene's test was used to evaluate homogeneity of variances. In case normality assumptions were violated, non‐parametric tests were applied. For baseline surveys (T1 and T3), variation in host community composition across sites was visualized using principal component analysis (PCA) on Hellinger‐transformed relative abundances of host colonies. Differences in community composition among sites and time points were tested using PERMANOVA, with site and time point as main effect and transect nested within site to account for variability at both levels (permutations = 999). Similarity Percentage (SIMPER) analysis was performed to investigate drivers of dissimilarity in community composition. Coral genus richness and effective diversity were assessed using Hill numbers: N1 (based on the exponential of the Shannon–Wiener index) and N2 (based on the inverse Simpson index). Differences between time points and among sites were tested using *t*‐tests and ANOVAs followed by Tukey's post hoc comparisons. For colonies with available diameter data, differences in colony size between time points were assessed using a Wilcoxon test. Changes in the proportion of fragmented coral colonies were assessed using either a chi‐square test or Fisher's exact test, depending on the sample size and distribution of expected frequencies. Fisher's exact test was applied when expected counts in any category (fragmented/unfragmented) fell below 5. Differences in crab abundance between time points were tested using a *t*‐test, while the change in prevalence (number of uninhabited and inhabited hosts) was analysed using a chi‐square test.

We modelled turnover rates derived from the fate‐tracking dataset of tagged colonies monitored across the four transitional periods (TR1–TR4) using a beta‐inflated (BEINF) regression model (Ospina and Ferrari 2010) to account for the high proportion of extreme values, specifically 0% (no turnover) and 100% (complete turnover) observed in our dataset. The BEINF distribution is suitable for such data as it combines a beta distribution for continuous values between 0 and 1 with Bernoulli components to model the probabilities of 0 and 1 values separately. In this model, turnover (*y*) was treated as a BEINF‐distributed random variable:

$$y\sim \text{BEINF}\left(\mu ,\sigma ,\upsilon ,\tau \right)$$

where μ mean turnover rate, σ represents the precision parameter controlling dispersion, υ represents the probability of zero inflation, τ represents the probability of one inflation.

Site and time point were included as fixed effects to assess whether turnover varied across reef locations or remained consistent over time. The model structure was as follows:

$$\mu_{i}={\text{logit}}^{-1}\left(\beta_{0}+\beta_{1}\times {\text{site}}_{i}+\beta_{2}\times {\text{time point}}_{i}\right)$$

$$\upsilon_{i}={\text{logit}}^{-1}\left(\alpha_{0}\right)$$

$$\tau_{i}={\text{logit}}^{-1}\left(\gamma_{0}\right)$$

where $\beta_{0}$, $\alpha_{0}$ and $\gamma_{0}$ are intercepts, and $\beta_{1}$ and $\beta_{2}$ represent the effects of site and time point respectively.

Background and disturbance‐based mortality rates were not modeled using BEINF due to poor predictive performance and small sample size. Instead, a permutation test procedure was developed and applied (Supporting Information—S1). Background mortality trends were examined by comparing means in a multi‐sample test, while disturbance‐based mortality was assessed by pooling pre‐ and post‐T2 (bleaching event) time points and testing for significant differences. For this analysis, we included colonies observed across all time points, or until mortality, restricting statistical analyses of turnover, background mortality, and disturbance‐based mortality to colonies inhabited by crabs at least once during the study period. This approach was applied uniformly across all sites, as host mortality is a natural outcome of the system and not experimentally controlled. Where relevant, changes in sample size were addressed by carefully considering their impact on result interpretation in the discussion.

## 3 Results

### 3.1 Gall Crab and Scleractinian Host Community

Seven coral families previously documented as cryptochirid hosts in the RS were observed within the transects, with Leptastreidae and Plesiastreidae being the only known hosts absent in this study. Associations were limited to *Fungia* within Fungiidae and *Pocillopora* within Pocilloporidae. Additionally, the crab genus *Neotroglocarcinus* was not observed. A complete list of all observed associations is provided in Table 1.

**FIGURE 2:**
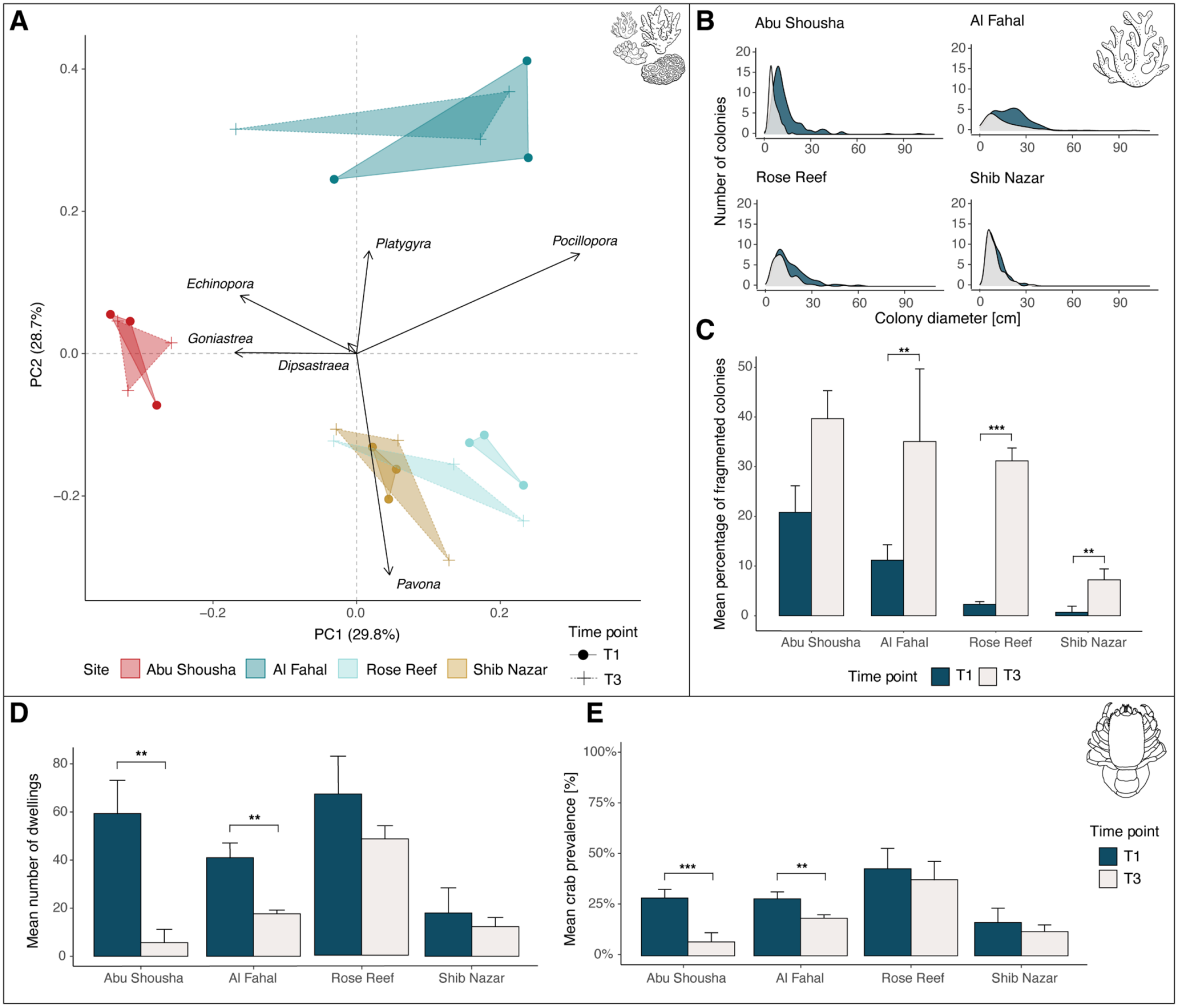
Coral host and cryptochirid community changes from baseline belt transect surveys (April 2023—May 2024). (A) Principal component analysis (PCA) of host species relative abundance across the four sites: Abu Shousha (red), Al Fahal (light blue), Rose Reef (dark blue), and Shib Nazar (tan), with three transects per site. Vectors represent the six most common coral genera, showing their contribution to community composition. Time points are differentiated by T1 (circles, solid lines) and T3 (crosses, dashed lines). (B) Density plots of host colony size for colonies with diameter data available. (C) Bar plot showing the mean percentages (with standard deviation) of fragmented colonies (colonies affected by partial mortality) for each transect at the four sites, compared by time point. (D) Mean number of dwellings and (E) Mean crab prevalence rates across the four sites for T1 and T3. Asterisks indicate significant differences (panels C and E: Chi‐square, panel D: *t*‐test; *p < 0.05, **p < 0.01, ***p < 0.001). Icons in the top right corners of panels indicate the data sources: Host community, individual colonies and gall crabs.

The coral host community composition differed significantly across sites (PERMANOVA, *p* < 0.001; Figure 2), with site explaining 66.7% of the variation and transects nested within sites contributing 21.0% (*p* < 0.001). SIMPER analysis highlighted *Pocillopora*, *Pavona*, and *Goniastrea* as the main genera driving site‐level differences, with variations most pronounced between Abu Shousha (AS) and other sites (Figure 2). Significant differences in effective diversity were observed across sites, with Rose Reef (RR) showing the highest values for both Hill numbers N1 and N2 (*p* < 0.001), while genus richness did not vary significantly across sites (Figure S2).

Colony size ranged from 10.4 ± 5.5 cm in maximum diameter at Shib Nazar (SN) to 20.9 ± 12.7 cm at Al Fahal (AF) (Figure 2). Colony fragmentation was more frequent at AS (20.8% ± 5.4%) and AF (11.2% ± 3.1%) compared to RR (2.3% ± 0.6%) and SN (0.7% ± 1.2%; Figure 2). Cryptochirid dwellings and prevalence mirrored these trends: RR recorded both the highest mean dwellings (67 ± 16) and prevalence (43.0% ± 10.0%), while SN had the lowest values (18 ± 10.4 dwellings; 16.4% ± 7.1% prevalence; Figure 2).

### 3.2 Baseline Shifts and Bleaching Impacts

Host community composition showed a significant effect of time point (*p* < 0.001); however, the interaction between site and time point was not significant (*p* = 0.89; Figure 2). SIMPER analysis revealed notable changes, with temporal shifts primarily driven by variations in the abundance of *Pocillopora*, *Pavona*, and *Echinopora*. Colony size decreased (the area of intact living tissue, not skeletal erosion) significantly at all sites (*p* < 0.001), except for SN (*p* = 0.09; Figure 2). Fragmentation increased markedly at AF, RR, and SN (all *p* < 0.01), with the largest rise observed at RR (31.1% ± 2.6%; Figure 2, Table S2).

Gall crab abundance and prevalence declined at AS and AF from T1 to T3. At AS, the number of dwellings dropped by 89.8% (*p* < 0.01), while AF experienced a 56.1% reduction (*p* < 0.01). Prevalence followed a similar trend, with significant declines at these sites (AS *p* < 0.001, AF *p* < 0.01, Figure 2). A decline for both variables was also observed at RR and SN, although not statistically significant (all *p* > 0.05, Tables S3–S5).

In 2022, peak temperatures ranged from 32.3°C to 32.9°C across study sites, occurring from late August to early September. In 2023, maximum temperatures increased to 32.9°C–33.4°C, peaking in late August. By 2024, peaks further rose to 33.4°C–34.0°C and were observed earlier, from early to mid‐August (Figure S3). In situ DHWs surpassed 4°C‐weeks at all sites from mid‐August to early September 2023 (nearshore to offshore), persisting until mid‐November to early December (offshore to midshore). By the study's end, temperatures rose earlier, with DHW exceeding eight at all sites except SN from mid‐July to early August (nearshore to offshore). Bleaching severity of tagged colonies varied across sites: RR recorded the highest prevalence (74.6%), followed by AF (55.0%), SN (47.4%), and AS (34.2%). Coral mortality was consistently high at AS, with 32.5% of host colonies lost during the bleaching period, compared to lower mortality rates at offshore sites (RR: 7.8%, SN: 19.3%). In September 2024 (T4), mortality remained high at AS (39.0%) and AF (29.3%), while offshore sites exhibited mortality rates below 11% (Figures S4 and S5; Tables S6–S13).

### 3.3 Rates of Change in Gall Crab Community

**FIGURE 3:**
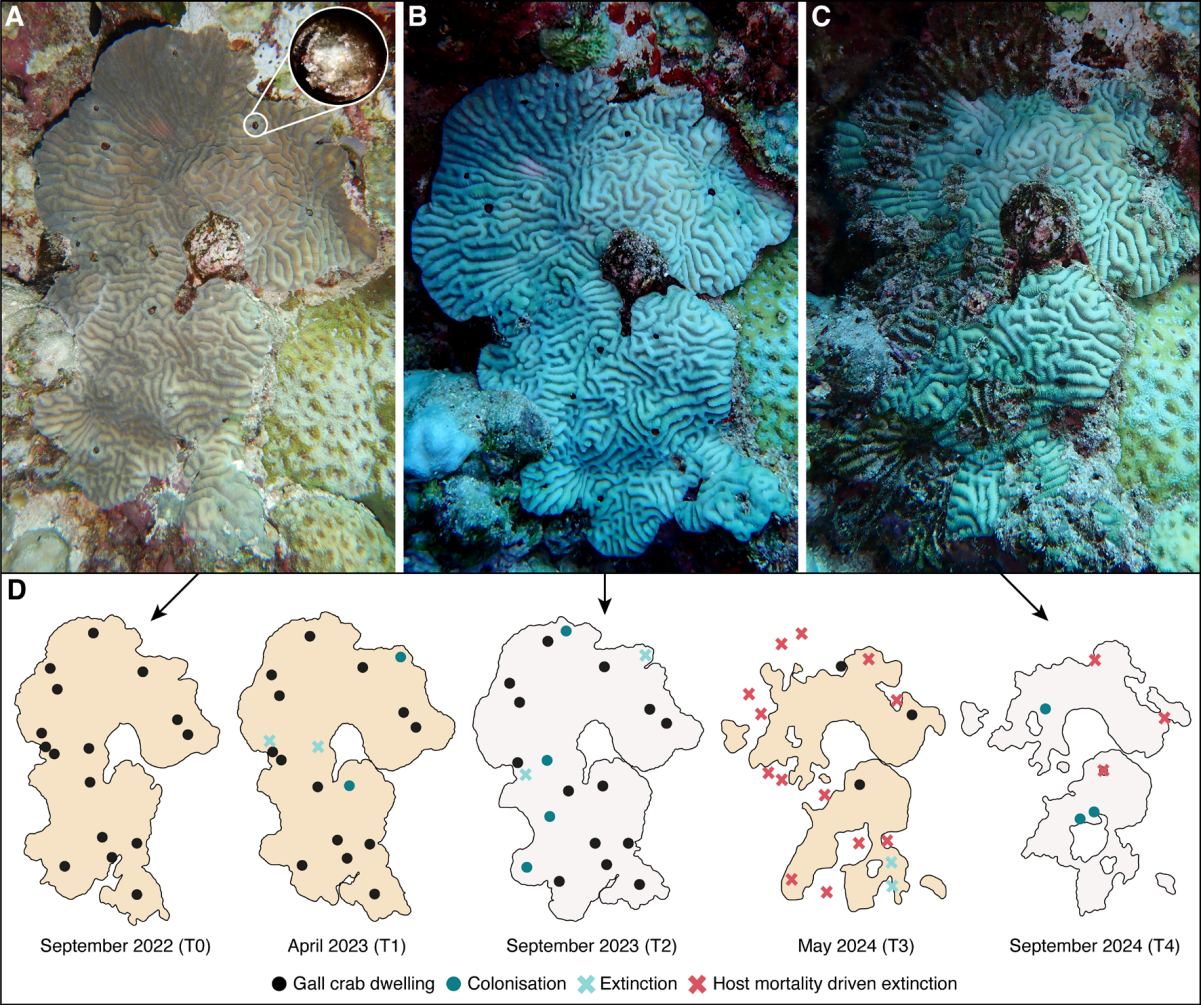
Gall crab turnover on a single host colony over the two‐year study period. (A) In situ photograph of a healthy *Platygyra lamellina* colony at RR in September 2022 (T0), initially inhabited by 16 gall crabs. Inset at the top right corner shows a close‐up photograph of a gall crab in its dwelling. (B) Photograph of the same colony in September 2023 (T2) in bleached condition, now with 18 dwellings. (C) The colony at the end of the study period in September 2024 (T4) with only three dwellings. (D) Schematic of the same colony at each time point (T0–T4), with black circles marking the 16 initial gall crab dwellings. Three different event types are indicated with colonizations = dark blue circles, extinctions = light blue crosses, and host mortality‐driven extinctions = red crosses. The colony's health status is represented by tan coloration for healthy tissue and white for bleached.

**FIGURE 4:**
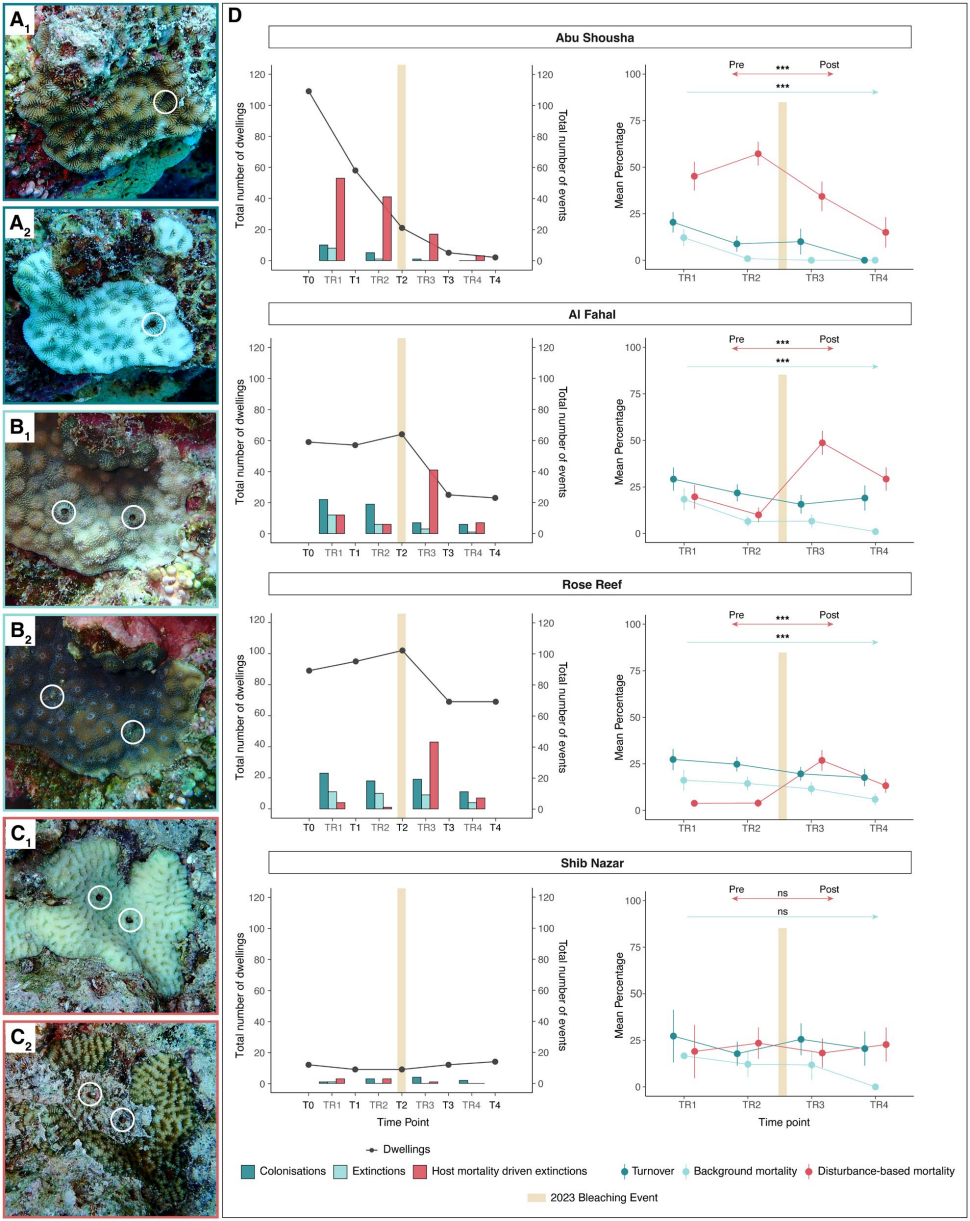
Impact of the 2023 bleaching event on the gall crab community over the course of the 2‐year study period. (A–C) Show examples of colonization (dark blue border), extinction (light blue border), and host mortality–driven extinction (red border) events at 6‐month intervals. (A1, A2) *Goniastrea pectinata* ; (B1, B2) *Echinopora gemmacea* ; (C1, C2) *Platygyra crosslandi* . White circles indicate the locations of dwellings or their previous positions. (D) Results of gall crab fate‐tracking. The left column shows total number of dwellings (grey points and line) at each time point (T0–T4) for the four reefs, with bars depicting the number of events: Colonizations (dark blue), extinctions (light blue), and host mortality–driven extinctions (red) at the transitional periods between time points (TR). Right column shows mean percentages and standard errors of the three rates (colonization, background mortality, and disturbance‐based mortality) calculated for each TR across the four sites. Arrows above the plotted rates represent results from the permutation test: Red arrows indicate significant differences in pre‐ and post‐disturbance means, and light blue arrows indicates significant trends in background mortality (*p < 0.05, **p < 0.01, ***p < 0.001, ns = not significant). The tan bar denotes the 2023 bleaching event.

Comparison of in situ photographs tracing individual gall crabs on coral colonies revealed three distinct event types: (1) Colonization, where a crab successfully established a new dwelling; (2) Extinction, where a dwelling was fully overgrown by the host colony; (3) Host mortality–driven extinction, where, following partial colony mortality, a dwelling remained visible on the dead coral skeleton but was empty (Figures 3, 4). Notably, primary colonizations, where previously uninhabited colonies became inhabited, were rare (Figure S6).

### 3.4 Gall Crab Population Dynamics Pre‐ and Post‐Disturbance

At the beginning of the study (T0), the total number of dwellings was highest at AS (109), followed by RR (89), AF (59), and SN (12). AS showed a continuous decline throughout the study, while AF and RR exhibited initial increases in dwellings until the 2023 bleaching event. SN, with the smallest crab population, showed minimal fluctuations over time (Figure 4).

Colonization consistently exceeded extinction at all sites and time points (Figure 4). Host mortality–driven extinction peaked post‐bleaching (TR3‐TR4), with 42.2% of the pre‐disturbance population lost at RR and 64.1% lost at AF from T2 to T3 (Figure 4). However, mortality subsequently returned to pre‐disturbance levels at both sites. At AS, host mortality–driven extinction was initially high (48.6% of the population lost till T1) and remained consistently elevated throughout the study (Figure 4). SN showed consistently low levels of colonization and extinction events throughout the study, reflecting the small population size at this site. However, colonization events peaked post‐bleaching at this site (Figure 4).

Cryptochirid turnover rates showed stability over the study period, except for a visible decline at TR4 in AS and SN (Figure 4). The BEINF model found no significant differences across time points, indicating no meaningful variation in turnover rates (all *p* > 0.05). However, the small sample size at TR4 for the site AS (*n* = 11) warrants caution in interpreting these findings. Turnover varied little between sites, with SN showing higher rates compared to the baseline site, AS (*p* < 0.05) (Figure 4). Although AF and RR also had higher turnover rates compared to AS, these differences were not statistically significant (*p* = 0.1 and *p* = 0.3, respectively). The BEINF model estimated a high zero‐inflation probability (*ν* = 0.9, *p* < 0.001), a moderate dispersion parameter (*σ* = 0.3, *p* < 0.001), and a one‐inflation probability of *τ* = 0.5 (*p* < 0.001).

In the transitional period TR1 (T0–T1), background mortality ranged from 18.4% ± 6.0% (SE) at AF to 12.1% ± 4.6% (SE) at AS (Figure 4). Permutation tests revealed significant declines in background mortality over time at AS (*p* < 0.01), AF (*p* < 0.01), and RR (*p* = 0.05) across the full study period (T0–T4), while SN remained unchanged (*p* = 0.20). Disturbance‐based mortality was initially low at AF (19.7% ± 6.4%), RR (3.8% ± 1.9%), and SN (19.0% ± 14.3%) but notably higher at AS (45.2% ± 7.7%), where it continued to increase until the bleaching event at T2, reaching 57.2% ± 6.3%. After T2, disturbance‐based mortality declined significantly at AS for the following transitional periods (TR3–TR4; *p* < 0.001), while it increased at AF and RR (*p* < 0.001 for both), reaching 48.7% ± 6.4% and 26.9% ± 5.5%, respectively. SN again showed no significant change (*p* = 0.83) (Figure 4; Tables S14, S15).

## 4 Discussion

### 4.1 Diversity and Abundance of Cryptochirid–Coral Associations

In this study, we provide the first comprehensive assessment of gall crab population dynamics along a cross‐shelf gradient in the central RS. Our findings on differences in coral host community composition (Figure 2) align with previous research, supporting the treatment of the four reefs as distinct ecological units (Khalil et al. 2017). Surveying all known hosts of Cryptochiridae at these four sites revealed a high diversity of hosts and crabs, highlighting cryptochirids as a common component on coral reefs in this region (Table 1). Our transect surveys revealed that crab abundance and prevalence broadly reflected the condition of their host communities (Figure 2). Sites with higher host diversity and colony size/density, such as RR, exhibited higher gall crab occurrence, whereas degraded reefs like SN (fewer and smaller coral colonies) or AS (lower host diversity: Hill N1 = 5.4 ± 1.0; N2 = 3.7 ± 0.8) showed lower occurrence values (Figure S2). This suggests that site‐specific effects and host density mediate gall crab distribution, consistent with density‐dependent recruitment patterns observed in other coral‐associated invertebrates (Preston and Doherty 1994; Rowley 2008; Britayev and Mikheev 2013). Overall, these spatial differences indicate that gall crab populations respond to reef‐specific conditions rather than following a clear cross‐shelf gradient. However, this study has some limitations, such as the exclusion of non‐host coral families (e.g., Acroporidae, Poritidae), resulting in a lack of coral cover data, uneven sampling areas, and a small number of surveyed reefs (*n* = 4).

### 4.2 Temporal Dynamics of Gall Crab Populations

Through the fate‐tracking of 799 individual crabs, this study provides novel insights into the temporal dynamics of these obligate coral‐dwellers (Figures 3, 4). Contrary to expectations of slow‐paced population variations associated with the occupation of large galls or deep pits in coral skeletons (Edmondson 1933; Bähr et al. 2023), we found high turnover rates characterized by consistent colonizations and extinctions across all study sites (Figure 4). Our findings reveal previously unknown variability in cryptochirid populations, while our documentation of lifespans for three genera (at least 2 years) confirms assumptions that gall crabs can persist in their hosts for extended periods (Simon‐Blecher and Achituv 1997; Kotb and Hartnoll 2002). The lack of comparable data on population‐level temporal dynamics in other coral‐dwelling decapods limits our ability to contextualize these findings. The most analogous system for which data is available is *Trapezia* crabs colonizing Pocilloporidae corals, where occurrence was found to correlate with host density and size but also to fluctuate based on season, reef, or variations in temperature (Gotelli et al. 1985; Britayev and Mikheev 2013; Canizales‐Flores et al. 2021; Merkin and Britayev 2023). Unlike cryptochirids, *Trapezia* crabs migrate between colonies, making individual tracking difficult (Castro 1978). Such mobility may introduce overlooked variability and likely results in underestimations of population turnover. By comparison, our findings suggest that symbiont‐coral systems may be far more dynamic than previously assumed. While adult migration can be ruled out for cryptochirids, factors such as larval supply may contribute to the observed patterns. However, little is known about larval mortality rates and settlement success in gall crabs, making it difficult to assess the role of larval supply variability in shaping population turnover. Future research should aim to integrate spatial population structure and connectivity analyses alongside long‐term monitoring to better understand the drivers of symbiont population dynamics in sessile organisms. Lastly, as a caveat, we note that re‐occupation of vacant dwellings could theoretically obscure extinctions or lead to overestimation of lifespans; however, there is currently no evidence that cryptochirids recolonize empty dwellings (see also Simon‐Blecher and Achituv 1997).

Colonizations consistently outnumbered extinctions, and primary colonizations were rare (Figure 4 and Figure S6). Additionally, the high zero‐inflation revealed by the BEINF model indicates that the large number of uninhabited colonies remained uninhabited, while the high one‐inflation suggests that inhabited colonies experienced full turnover. These patterns indicate a strong tendency to settle on already inhabited hosts, contrasting with the hypothesis that gall crab larvae do not exhibit settlement preferences based on the presence of adult crabs (Nogueira et al. 2014). Settlement preferences for already inhabited hosts are a trend observed in symbiotic *Spirobranchus* polychaetes (e.g., Rowley 2008) and have also been documented in other decapods such as the pea crab *Dissodactylus crinitichelis* Moreira, 1901, where the presence of conspecifics on a host ( *Encope emarginata* (Leske, 1778)) strongly influenced settlement behavior and mating success (Souza et al. 2019). However, the underlying mechanisms driving cryptochirid settlement, such as potential cues and larval behavior, remain unclear and warrant further investigation. Lastly, corals overgrew empty dwellings, and new, sizable dwellings were formed within 6 months, supporting the assumption that cryptochirids actively contribute to dwelling formation and maintenance (Figures 3 and 4; Simon‐Blecher and Achituv 1997).

Our results align with our hypothesis that transect surveys, while effective in identifying spatial patterns, significantly underestimate temporal population variations. Steady colonization and background mortality (both host and cryptochirid) would likely offset one another in transect surveys, obscuring the true extent of population turnover. Turnover rates exhibited limited spatial variation, though small sample sizes due to host mortality reduced the statistical power to detect finer spatial heterogeneity. SN—the most sparsely populated site—showed unexpectedly high turnover, suggesting that even small populations can sustain reproductively active populations or successfully recruit (Figure 4). These findings contrast with motile decapods like *Trapezi*a, where host density and size strongly shape colonization rates, emphasizing the need for further research into environmental and ecological drivers of coral‐associated decapod population dynamics (Britayev and Mikheev 2013; Canizales‐Flores et al. 2021; Merkin and Britayev 2023).

### 4.3 Disturbance Impact on Gall Crab Population Dynamics

In 2023, coral communities at our study sites experienced significant thermal stress, with intensity varying nearshore to offshore. Host coral bleaching severity followed a cross‐shelf gradient, with nearshore sites suffering higher host mortality, consistent with patterns observed in previous events in this region (Figure S3; Furby et al. 2013, Monroe et al. 2018). Baseline surveys revealed significant declines in crab abundance at nearshore sites but limited changes at the two offshore sites, demonstrating population resilience despite the occurrence of bleaching (Figure 2). This likely reflects the lower levels of host mortality (partial or full) offshore, indicating that cryptochirid mortality was not a direct result of elevated temperatures and bleaching, but instead followed the mortality of their coral hosts. Bleaching and subsequent mortality are known to restructure coral‐associated communities, with obligate symbionts declining and being replaced by opportunistic or facultative species on dead coral heads (Enochs and Manzello 2012; Salas‐Moya et al. 2021). Consistent with these findings, gall crabs perish when their host colonies die. However, they exhibit greater resilience on bleached colonies, persisting longer than motile symbionts, like *Trapezia*, which often migrate or suffer sharp population declines even before host mortality occurs (Tsuchiya et al. 1992; Stella, Munday et al. 2011; Stella et al. 2014).

Fate‐tracking revealed consistent colonization across most sites despite disturbance (Figures 3 and 4). However, at AS, gall crab populations experienced a population collapse, driven by multiple stressors, including bleaching and predation by Crown‐of‐Thorns Starfish (CoTS). Over the two‐year time span, gall crab numbers on tagged colonies at this site plummeted from nearly 120 individuals to below five. Frequent observations of CoTS on transects and in the surrounding area (25 specimens on 30 min transect; Nunes Peinemann, personal communication) suggest they may play a considerable role in this decline. Initially, CoTS predation caused high disturbance–driven mortality. However, as host colonies and gall crab populations decreased, mortality rates declined simply because few individuals were left. This reversal underscores how compounded stressors can cascade through ecosystems, disrupting population dynamics and community stability, a phenomenon frequently discussed in ecology research (Côté et al. 2016; Froehlich et al. 2021).

While crab turnover rates showed limited spatial variation, distinct mortality patterns emerged across sites. SN, despite its low host density and small colony size, exhibited negligible background and disturbance‐driven mortality (Figure 4). RR and AF showed similar resilience, maintaining population stability post‐disturbance despite differing levels of host mortality (Figure 4). Consistent colonizations across all sites except for AS underscore the high reproductive output and ongoing successful recruitment of gall crabs, even under impaired host conditions. This resilience contrasts with the reduced fecundity and population declines observed in *Trapezia* crabs on bleached hosts (e.g., Stella et al. 2014).

### 4.4 Ecological Significance and Future Directions

This study provides novel insights into coral‐dwelling decapod biology and their ecological roles on coral reefs, highlighting for gall crabs: (1) high individual turnover, (2) settlement preference for inhabited colonies, (3) resilience through reproduction on bleached hosts, allowing populations to buffer short‐term disturbances, and (4) vulnerability to host mortality. By considering these findings alongside gall crabs' unique lifehistory traits, we can better understand the challenges they pose to established ecological frameworks. While barnacles align well with the r/K‐selection framework (Brickner et al. 2010), cryptochirids seem to challenge these classifications by combining traits of both r‐ and K strategists (Bähr et al. 2021). Our findings reinforce this duality; the significant temporal variability in cryptochirid populations reflects their investment in reproduction, despite small populations. Additionally, their settlement behavior reflects a K‐selected process, as they not only target specific coral genera or species but also prefer previously colonized hosts. These density‐dependent dynamics underscore the limitations of the r/K‐selection framework, as highlighted by Reznick et al. (2002), while also illustrating the evolution of a highly specialized symbiotic relationship. The positive feedback loop of high reproductive investment to offset larval mortality linked to host specificity and settlement preferences likely drove the evolution of reproductive adaptations in cryptochirids, such as the development of a brood pouch (marsupium) or the capability to store sperm from multiple matings (Vehof et al. 2016). These strategies highlight how gall crabs balance host dependency with maximizing reproductive success, likely contributing to their observed resilience.

The ecological roles of small dwelling (in)vertebrates within coral reef ecosystems are not yet well understood. Brandl et al. (2019) identified nutrient cycling and secondary production as essential processes underpinning reef functionality when studying the role of cryptobenthic reef fishes, which occupy a similar trophic guild to gall crabs. These fish exemplify these roles by consuming resources inaccessible to larger consumers and contributing energy back to the reef system through their high turnover rates and larval supply, which serve as critical energy sources for higher trophic levels (Brandl et al. 2018). Gall crabs, however, lack a well‐documented trophic link to higher levels due to minimal observed predation (Leray et al. 2015). By consuming coral mucus, they transform host‐derived resources into energy potentially accessible to other reef organisms—a process hypothesized not only for gall crabs but also for other coral‐associated invertebrates such as epizoic worms that exploit coral mucus as a trophic resource (Naumann et al. 2010; Bravo et al. 2024). Additionally, their larvae potentially contribute to zooplankton communities, providing an indirect link to higher trophic levels. While direct evidence of predation within the cryptic guild remains limited, background mortality rates hint at possible trophic interactions that warrant further investigation. By examining turnover and mortality, this study provides measurable indicators of change that Streit and Bellwood (2023) emphasize as “rate traits”—dynamic and quantifiable characteristics that are essential for linking biodiversity to ecosystem functions. Connecting these traits to reproductive strategies, this study establishes a foundation for future research into the functional roles of coral‐associated invertebrates.

In conclusion, this study provides the first comprehensive assessment of temporal variation in cryptochirid populations, shedding light on their biology, resilience, and ecological roles. Our findings of unexpectedly high turnover, population resilience at most sites despite extensive bleaching, and vulnerability to host coral mortality emphasize the evolution of a highly specialized symbiotic relationship shaped by mechanisms such as settlement preferences, host dependency, and reproductive strategies. The population collapse at AS, driven by multiple simultaneous disturbances, illustrates the limits of resilience when host mortality crosses critical thresholds. These results, coupled with the increasing frequency of coral bleaching events—exemplified by the onset of another major bleaching event at the end of our study period—underscore the necessity of maintaining sufficient coral cover to support the survival of these obligate symbionts. Currently, gall crabs and other obligate symbionts remain overlooked in conservation efforts. While many host coral species have been assessed for their conservation status, symbiotic coral fauna have yet to receive similar attention (Bravo et al. 2022). By demonstrating how population dynamics of coral‐associated organisms are directly impacted by disturbances, this study underscores the urgent need to integrate these symbionts into conservation strategies to protect reef biodiversity and sustain ecosystem functionality in the face of accelerating environmental change.

## Author Contributions

**Susanne Bähr:** conceptualization (equal), data curation (lead), formal analysis (equal), investigation (lead), methodology (lead), project administration (equal), visualization (lead), writing – original draft (lead). **Natalie Dunn:** conceptualization (supporting), data curation (supporting), investigation (supporting), resources (supporting), writing – review and editing (supporting). **Sancia E. T. van der Meij:** investigation (supporting), supervision (equal), validation (equal), writing – review and editing (equal). **Joydeep Chowdhury:** formal analysis (equal), methodology (supporting), software (supporting), validation (supporting). **Francesca Benzoni:** conceptualization (equal), funding acquisition (lead), project administration (equal), resources (lead), supervision (equal), validation (equal), writing – review and editing (equal).

## Conflicts of Interest

The authors declare no conflicts of interest.

## Supporting information


**Data S1.** Supporting Information.

## Data Availability

Data and code are available on figshare: https://figshare.com/s/d22e3c292bb61de9d056.
