# Supplementary material for: Temporal Dynamics and Disturbance Responses in Coral‐Dwelling Decapods Provide a Novel Perspective on Their Ecological Role in Coral Reef Systems
Source: Ecol Evol. 2025 Jun 3;15(6):e71474. doi: 10.1002/ece3.71474 (PMC12134086; doi:10.1002/ece3.71474)
Supplement: Supplementary file 1 — Data S1. Supporting Information. [file ECE3-15-e71474-s001.docx]

**Supplemental Information for:**

**Temporal Dynamics and Disturbance Responses in Coral-Dwelling Decapods Provide a Novel Perspective on Their Ecological Role in Coral Reef Systems**

**Table of Contents:**

| **Sup Figure 1** | Page 2 |
| --- | --- |
| **Sup Figure 2** | Page 3 |
| **Sup Figure 3** | Page 4 |
| **Sup Figure 4** | Page 5 |
| **Sup Figure 5** | Page 6 |
| **Sup Figure 6** | Page 7 |
| **Permutation test procedure** | Page 8 |
| **Sup Table 1** | Page 9 |
| **Sup Table 2-5** | Page 10 |
| **Sup Table 6-11** | Page 11 |
| **Sup Table 12-15** | Page 12 |


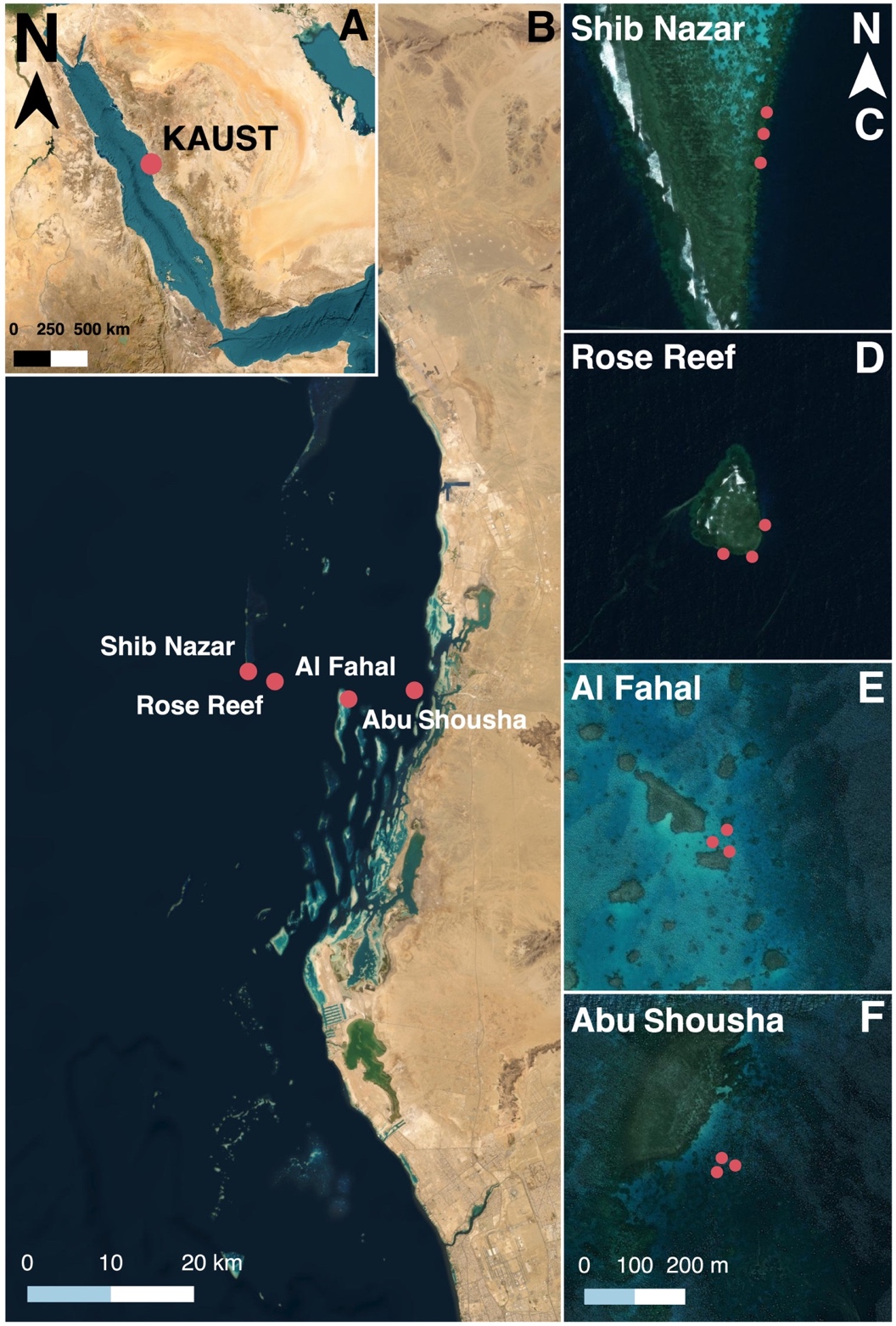
\

**Sup Fig 1**: **Map of the study area in the central Saudi Arabian Red Sea, showing the four study sites.** A) Inset showing the location of the study sites within the Red Sea, with King Abdullah University of Science and Technology (KAUST) marked as a reference point. B) Main map with the four study sites (Shib Nazar, Rose Reef, Al Fahal, and Abu Shousha) indicated by red circles. C-F) Panels on the right provide enlarged views of each site, with circles representing the three transects at each location.


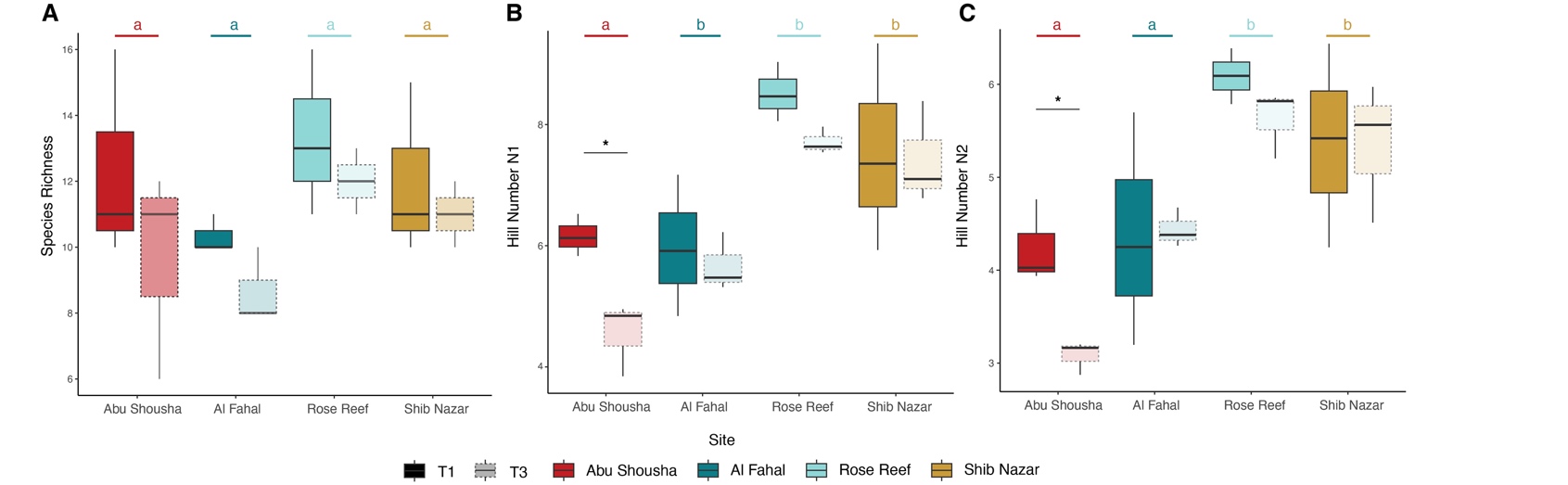


**Sup Figure 2: Boxplots showing species richness and diversity indices based on April 2023 and May 2024 baseline transect surveys.** A) Species richness, B) Hill number N1 (Shannon-Wiener index), and C) Hill Number N2 (Simpson index) for the four study sites at two-time points (T1 and T3). Sites are coloured as follows: Abu Shousha (red), Al Fahal (light blue), Rose Reef (dark blue), and Shib Nazar (tan). T1 is represented by solid bars, and T3 by dashed bars with more transparent fills. Solid black bars with asteriks above the boxplots indicate significant differences between time points, as determined by t-tests (p < 0.05*, p < 0.01**, p < 0.001***). Bars with letters colour coded by site above the boxplots represent significant differences between sites based on ANOVA results; the same letter indicates no significant difference between groups.

**Sup Figure 3** **Daily average temperatures (°C, upper lines) and Degree Heating Weeks (DHW, lower lines) from August 2022 to September 2024 for four reefs in the central Red Sea.** Temperature data (n = 3 loggers per reef) are shown for Abu Shousha (red), Al Fahal (dark blue), Rose Reef (light blue), and Shib Nazar (tan). NOAA SST data were retrieved from the Medina/Makkah NOAA station (grey line) from <https://coralreefwatch.noaa.gov/product/5km/index.php>. The horizontal grey line indicates the coral bleaching threshold (31.9°C) based on Monthly Maximum Mean (MMM) + 1°C based on NOAA climatology (1985–2012). DHW values (°C-weeks, red secondary y-axis) were calculated using in situ temperatures and same NOAA climatology. Dashed red lines mark the 4°C-weeks and 8°C-weeks thresholds, indicating expected reef-wide bleaching and mortality of heat-sensitive corals, respectively as defined by Coral Reef Watch (CRW). Sampling time points (T0–T4) of this study are indicated on the x-axis.


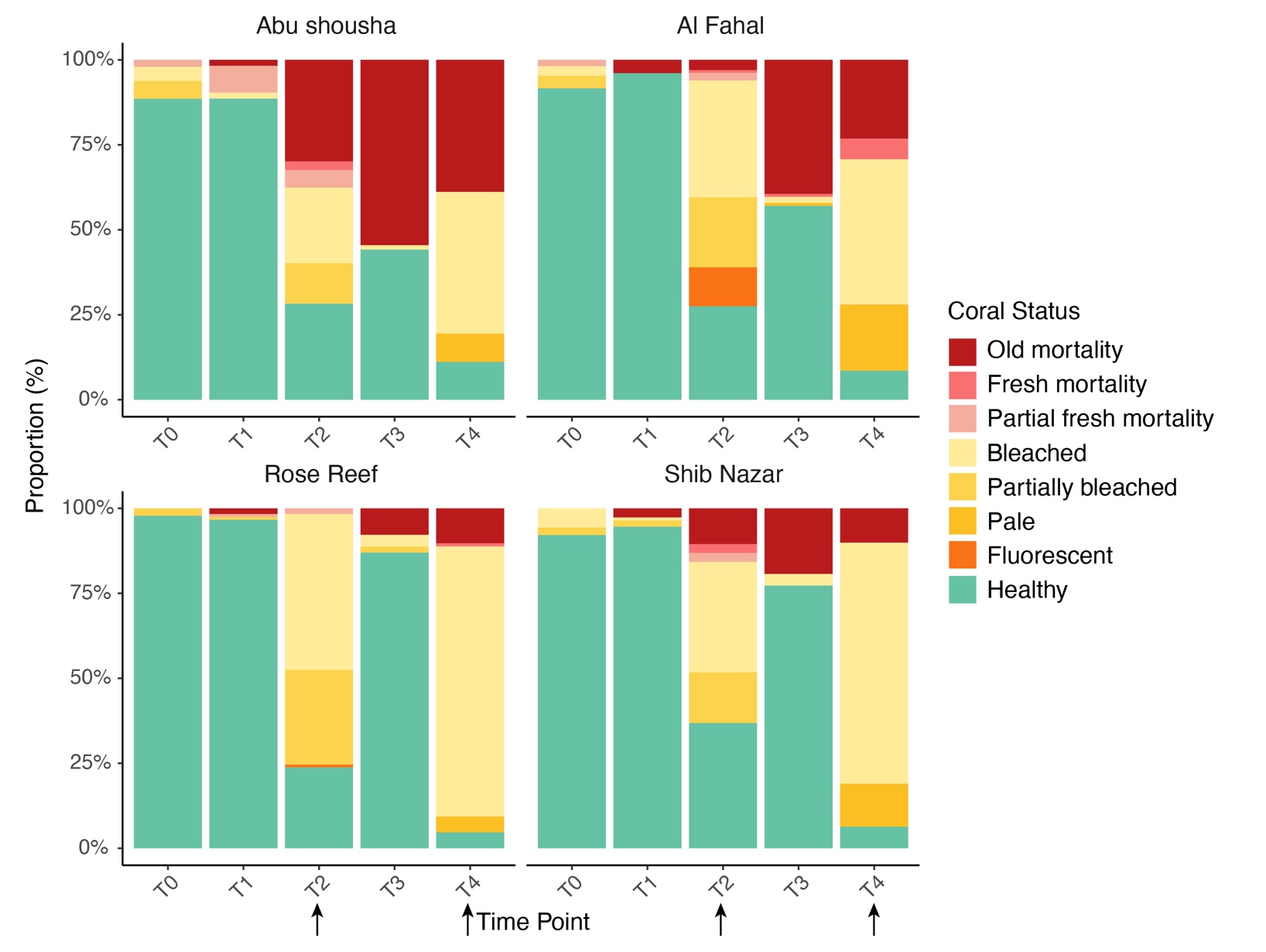


**Sup Figure 4: Impact of two coral bleaching events on coral health conditions of tagged coral colonies.**
Stacked bar plot showing the proportion of coral health statuses for tagged colonies across four sites (Abu Shousha, Al Fahal, Rose Reef, and Shib Nazar) at five time points (T0 to T4), scaled to 100%. Arrows indicate the occurrence of the two bleaching events at T2 and T4.

**
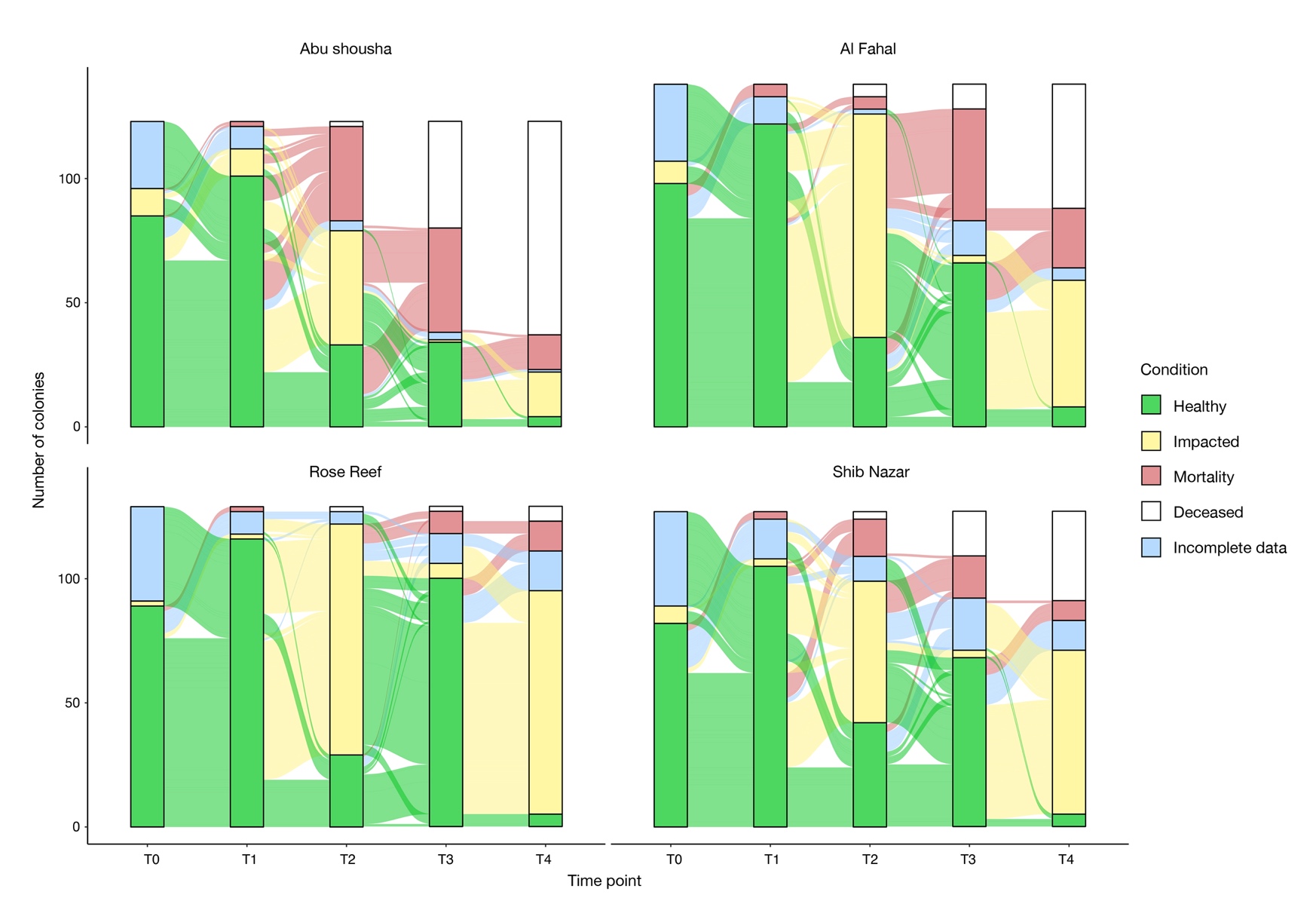
**

**Sup Figure 5**: **Alluvial plot showing the fate of tagged colonies throughout the study period (Sep 2023-2024).** The plot depicts the transitions in coral health status across the four sites over the study period. Each bar represents the number of colonies at each time point (T0–T4), with the colours indicating coral condition: **green** = healthy corals, showing no signs of partial/full bleaching, paling, or fluorescence; **yellow** = impacted corals, exhibiting signs of bleaching (partial/full), paling, fluorescence, or partial fresh mortality; **red** = mortality, indicating colonies that experienced full mortality (either old or fresh); **white** = represents colonies, those that died in the previous time point and were removed from the dataset; **blue** = incomplete data, where gall crab data for some colonies were unavailable. The flow of colours between time points highlights transitions in coral health.


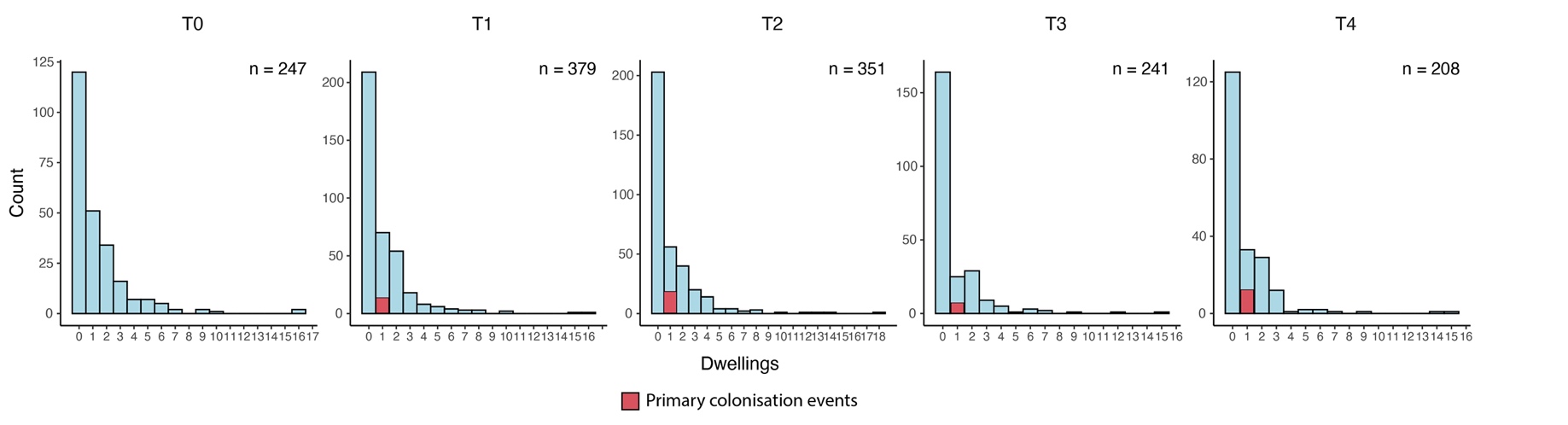


**Sup Figure 6: Histograms showing the number of gall crab dwellings on tagged host colonies.**
The histograms display the count of colonies and the number of cryptochirid dwellings (blue bars). The red bars within the "1" bin indicate primary colonisation events, representing the number of new gall crab dwellings established between time points. The total number of colonies (n) surveyed for each time point is indicated above each histogram.

**Permutation test procedure**

Let the two samples be denoted as D1 and D2, respectively, and the corresponding means of the samples be $d_{1}$ and $d_{2}$, respectively. The test statistic for the two-sample permutation test for a difference in means is $|d_{1}-d_{2}|$. We concatenate the two samples and randomly permute the observations in the combined sample, and then partition the permuted combined sample into the two groups with their sizes being the same as the original samples. That is, we partition the permuted combined sample into two groups D1* and D2* such that the size of D1 is the same as D1* and the size of D2* is the same as D2. For each such random permutation and subsequent partition, let the statistic obtained from the permuted samples D1* and D2* be $\left| {d^{*}}_{1}-{d^{*}}_{2} \right|$, where ${d^{*}}_{1}$ and ${d^{*}}_{2}$ are the means of D1* and D2*, respectively. The collection of $\left| {d^{*}}_{1}-{d^{*}}_{2} \right|$ values for all possible permutations of the combined sample gives the permutation null distribution of the test statistic. But because the total number of permutations is very large, we use 1000 random permutations. The permutation p-value is given by the proportion of values of $\left| {d^{*}}_{1}-{d^{*}}_{2} \right|$ larger than $|d_{1}-d_{2}|$.

Let the samples for the time points T0, T1, T2, T3 and T4 be denoted as S0, S1, S2, S3 and S4, respectively, and the corresponding means of the samples be $m_{0}$, $m_{1}$, $m_{2}$, $m_{3}$ and $m_{4}$, respectively. The test statistic for the permutation test for a decreasing trend in the means is $\sum_{i=0}^{3} (m_{i}-m_{i+1})$. To approximate the permutation null distribution of this statistic, we concatenate all the samples, randomly permute the observations in the combined sample, and then partition the permuted combined sample into the respective sizes of the original samples. That is, we partition the permuted combined sample into S0*, S1*, S2*, S3* and S4* such that the size of S0 is the same as S0*, the size of S1* is the same as S1, and so on. For each such random permutation and subsequent partition, let the statistic obtained from the permuted samples S0*, S1*, S2*, S3* and S4* be $\sum_{i=0}^{3} \left( {m^{*}}_{i}-{m^{*}}_{i+1} \right)$, where the ${m_{i}}^{*}'$s are the means of the permuted samples. The collection of $\sum_{i=0}^{3} \left( {m^{*}}_{i}-{m^{*}}_{i+1} \right)$ values for all possible permutations of the combined sample gives the permutation null distribution of the test statistic. But because the total number of permutations is very large, we use 1000 random permutations. The permutation p-value is given by the proportion of values of $\sum_{i=0}^{3} \left( {m^{*}}_{i}-{m^{*}}_{i+1} \right)$ larger than $\sum_{i=0}^{3} (m_{i}-m_{i+1})$.

| **Time Point** | **Date** | **Site** | **Lat** | **Long** | **Transect** | **Depth (m)** | **Area covered (m2)** | **Colonies recorded (n)** | **Density (n/m2)** |
| --- | --- | --- | --- | --- | --- | --- | --- | --- | --- |
| T1 | 12.04.23 | AS | N22.302616 | E39.048571 | 1 | 9 | 9.5 | 78 | 8.2 |
| T1 | 12.04.23 | AS | N22.302492 | E39.048519 | 2 | 9 | 5.25 | 116 | 22.1 |
| T1 | 12.04.23 | AS | N22.302568 | E39.048817 | 3 | 9 | 3.45 | 71 | 20.6 |
| T1 | 08.05.23 | AF | N22.295028 | E38.972117 | 4 | 5 | 10 | 66 | 6.6 |
| T1 | 08.05.23 | AF | N22.294813 | E38.971924 | 5 | 5 | 10 | 66 | 6.6 |
| T1 | 08.05.23 | AF | N22.294634 | E38.972168 | 6 | 5 | 10 | 51 | 5.1 |
| T1 | 11.04.23 | SN | N22.324203 | E38.856570 | 7 | 9 | 10 | 60 | 6.0 |
| T1 | 11.04.23 | SN | N22.324203 | E38.856570 | 8 | 9 | 10 | 51 | 5.1 |
| T1 | 11.04.23 | SN | N22.323379 | E38.856442 | 9 | 9 | 10 | 47 | 4.7 |
| T1 | 05.04.23 | RR | N22.310118 | E38.886224 | 10 | 8 | 3.2 | 68 | 21.3 |
| T1 | 06.04.23 | RR | N22.310608 | E38.886460 | 11 | 8 | 5.5 | 51 | 9.3 |
| T1 | 06.04.23 | RR | N22.310136 | E38.885690 | 12 | 8 | 3.65 | 53 | 14.5 |
| T3 | 08.05.24 | AS | N22.302616 | E39.048571 | 1 | 9 | 10 | 47 | 4.7 |
| T3 | 08.05.24 | AS | N22.302492 | E39.048519 | 2 | 9 | 5.4 | 73 | 13.5 |
| T3 | 08.05.24 | AS | N22.302568 | E39.048817 | 3 | 9 | 3.6 | 45 | 12.5 |
| T3 | 07.05.24 | AF | N22.295028 | E38.972117 | 4 | 5 | 10 | 49 | 4.9 |
| T3 | 07.05.24 | AF | N22.294813 | E38.971924 | 5 | 5 | 10 | 55 | 5.5 |
| T3 | 07.05.24 | AF | N22.294634 | E38.972168 | 6 | 5 | 10 | 45 | 4.5 |
| T3 | 09.05.24 | SN | N22.324203 | E38.856570 | 7 | 9 | 10 | 75 | 7.5 |
| T3 | 09.05.24 | SN | N22.324203 | E38.856570 | 8 | 9 | 10 | 57 | 5.7 |
| T3 | 09.05.24 | SN | N22.323379 | E38.856442 | 9 | 9 | 10 | 65 | 6.5 |
| T3 | 06.05.24 | RR | N22.310118 | E38.886224 | 10 | 8 | 3.4 | 98 | 28.8 |
| T3 | 06.05.24 | RR | N22.310608 | E38.886460 | 11 | 8 | 5.75 | 60 | 10.4 |
| T3 | 06.05.24 | RR | N22.310136 | E38.885690 | 12 | 8 | 3.6 | 55 | 15.3 |

**Table 1 Baseline transect survey metadata**

**Table 2 Chi square test for differences in fragmented colonies between T1 and T3**

| **Site** | **Chi_squared_stat** | **p_value** |
| --- | --- | --- |
| Abu Shousha | 1.05217 | 0.30501 |
| Al Fahal | 9.61538 | 0.00193** |
| Rose Reef | 38.20755 | 0.00000*** |
| Shib Nazar | 8.33333 | 0.00389** |

**Table 3 Descriptive statistics (mean and standard deviation) for gall crab prevalence and number of dwellings**

| **Site** | **Time_Point** | **mean_proportion** | **sd_proportion** | **mean_dwellings** | **sd_dwellings** |
| --- | --- | --- | --- | --- | --- |
| Abu Shousha | T1 | 0.285 | 0.042 | 59 | 13.8 |
| Abu Shousha | T3 | 0.068 | 0.046 | 6 | 5.5 |
| Al Fahal | T1 | 0.281 | 0.034 | 41 | 6.1 |
| Al Fahal | T3 | 0.185 | 0.017 | 18 | 1.5 |
| Rose Reef | T1 | 0.430 | 0.100 | 67 | 15.7 |
| Rose Reef | T3 | 0.376 | 0.090 | 48 | 5.5 |
| Shib Nazar | T1 | 0.164 | 0.071 | 18 | 10.4 |
| Shib Nazar | T3 | 0.119 | 0.034 | 12 | 3.8 |

**Table 4 Results of t-test for differences in number of dwellings between T1 and T3**

| **Site** | **Variable** | **T_Value** | **P_Value** |
| --- | --- | --- | --- |
| Abu Shousha | Total Dwellings | 6.257 | 0.003** |
| Al Fahal | Total Dwellings | 6.444 | 0.003** |
| Rose Reef | Total Dwellings | 1.941 | 0.124 |
| Shib Nazar | Total Dwellings | 0.884 | 0.427 |

**Table 5 Results of chi square test for differences in crab prevalence between T1 and T3**

| **Site** | **Variable** | **X-squared** | **P_Value** |
| --- | --- | --- | --- |
| Abu Shousha | Prevalence | 46.545 | 0.000*** |
| Al Fahal | Prevalence | 9.720 | 0.002** |
| Rose Reef | Prevalence | 0.968 | 0.353 |
| Shib Nazar | Prevalence | 1.884 | 0.170 |

**Table 6 t-test genus richness differences between T1 and T3**

| **site** | **shapiro_p** | **t_test_p** | **t_test_statistic.t** | **mean_time1** | **mean_time2** |
| --- | --- | --- | --- | --- | --- |
| Abu Shousha | 0.65857912 | 0.36708617 | 1.016001016 | 12.33333333 | 9.666666667 |
| Al Fahal | 0.10117066 | 0.11314515 | 2.236067977 | 10.33333333 | 8.666666667 |
| Rose Reef | 0.19476782 | 0.46469536 | 0.852802865 | 13.33333333 | 12 |
| Shib Nazar | 0.07964145 | 0.59033182 | 0.612372436 | 12 | 11 |

**Table 7 t-test Simpson index differences between T1 and T3**

| **site** | **shapiro_p** | **t_test_p** | **t_test_statistic.t** | **mean_time1** | **mean_time2** |
| --- | --- | --- | --- | --- | --- |
| Abu Shousha | 0.66692715 | 0.00792665 | 4.978240825 | 0.767110946 | 0.669534162 |
| Al Fahal | 0.29507845 | 0.71699915 | -0.41480159 | 0.761871626 | 0.779849537 |
| Rose Reef | 0.44524102 | 0.1875806 | 1.637104546 | 0.843169294 | 0.829111825 |
| Shib Nazar | 0.47650859 | 0.93791959 | -0.083489725 | 0.814836731 | 0.817314223 |

**Table 8 t-test Shannon-Wiener index differences between T1 and T3**

| **site** | **shapiro_p** | **t_test_p** | **t_test_statistic.t** | **mean_time1** | **mean_time2** |
| --- | --- | --- | --- | --- | --- |
| Abu Shousha | 0.62619469 | 0.046620248 | 3.553973058 | 1.815599461 | 1.510712076 |
| Al Fahal | 0.96266181 | 0.759539038 | 0.338517916 | 1.773788336 | 1.732540298 |
| Rose Reef | 0.63548115 | 0.078149607 | 2.66351254 | 2.136321411 | 2.038836524 |
| Shib Nazar | 0.95753179 | 0.986005021 | 0.01908448 | 1.999741139 | 1.996979593 |

**Table 9 ANOVA for genus richness site comparison**

| **term** | **df** | **sumsq** | **meansq** | **statistic** | **p.value** |
| --- | --- | --- | --- | --- | --- |
| site | 3 | 31 | 10.33333333 | 2.190812721 | 0.120773236 |
| Residuals | 20 | 94.3333333 | 4.716666667 |  |  |

**Table 10 ANOVA for Simpson index site comparison**

| **term** | **df** | **sumsq** | **meansq** | **statistic** | **p.value** |
| --- | --- | --- | --- | --- | --- |
| site | 3 | 0.04935815 | 0.016452715 | 9.572826262 | 0.000398098 |
| Residuals | 20 | 0.03437379 | 0.001718689 |  |  |

**Table 11 ANOVA for Shannon-Wiener index site comparison**

| **term** | **df** | **sumsq** | **meansq** | **statistic** | **p.value** |
| --- | --- | --- | --- | --- | --- |
| site | 3 | 0.720769399 | 0.240256466 | 11.35531863 | 0.000144596 |
| Residuals | 20 | 0.423161118 | 0.021158056 |  |  |

**Table 12 Tukey post hoc Simpson index site comparison**

| **comparison** | **diff** | **lwr** | **upr** | **p adj** |
| --- | --- | --- | --- | --- |
| Al Fahal-Abu Shousha | 0.05253803 | -0.0144552 | 0.11953128 | 0.15878583 |
| Rose Reef-Abu Shousha | 0.11781801 | 0.05082475 | 0.18481126 | 0.000441936 |
| Shib Nazar-Abu Shousha | 0.09775292 | 0.03075967 | 0.16474618 | 0.002986453 |
| Rose Reef-Al Fahal | 0.06527998 | -0.0017133 | 0.13227323 | 0.057820614 |
| Shib Nazar-Al Fahal | 0.0452149 | -0.0217784 | 0.11220815 | 0.264044817 |
| Shib Nazar - Rose Reef | -0.0200651 | -0.0870583 | 0.04692817 | 0.835642702 |

**Table 13 Tukey post hoc Shannon-Wiener index site comparison**

| **comparison** | **diff** | **lwr** | **upr** | **p adj** |
| --- | --- | --- | --- | --- |
| Al Fahal-Abu Shousha | 0.09000855 | -0.1450469 | 0.325063954 | 0.710121941 |
| Rose Reef-Abu Shousha | 0.4244232 | 0.18936779 | 0.659478605 | 0.000328108 |
| Shib Nazar-Abu Shousha | 0.3352046 | 0.10014919 | 0.570260003 | 0.003686208 |
| Rose Reef-Al Fahal | 0.33441465 | 0.09935924 | 0.569470057 | 0.003765793 |
| Shib Nazar-Al Fahal | 0.24519605 | 0.01014064 | 0.480251455 | 0.038970189 |
| Shib Nazar - Rose Reef | -0.0892186 | -0.324274 | 0.145836804 | 0.715583848 |

**Table14 Permutation test results for disturbance-based mortality**

| **Site** | **Comparison** | **p-value** |
| --- | --- | --- |
| Abu shousha | TR1/TR2 - TR3/TR4 | 0.001*** |
| Al Fahal | TR1/TR2 - TR3/TR4 | 0*** |
| Rose Reef | TR1/TR2 - TR3/TR4 | 0*** |
| Shib Nazar | TR1/TR2 - TR3/TR4 | 0.826 |

**Table 15 Permutation test results for background mortality**

| **Site** | **Comparison** | **p-value** |
| --- | --- | --- |
| Abu shousha | Trend TR1-TR4 | 0.004 |
| Al Fahal | Trend TR1-TR4 | 0.005 |
| Rose Reef | Trend TR1-TR4 | 0.05 |
| Shib Nazar | Trend TR1-TR4 | 0.203 |
